# Assessing the precision of 3D-printed surgical templates in oral implant placement: a comparison of half and full-guided approaches

**DOI:** 10.3389/fdmed.2025.1700363

**Published:** 2025-11-26

**Authors:** Chengmo Lin, Baohui Su, Yunli Chen, Wei Wang, Fujiang He, Yue Lan, Ling Jing

**Affiliations:** 1 College of Biomedical Engineering, Sichuan University, Chengdu, China; 2 Guangyuan Stomatological Hospital, Guangyuan, China

**Keywords:** half-guided surgical template, fully guided surgical template, digital surgical template, accuracy, oral implantology, bone density

## Abstract

**Background:**

Digital guided implantology improves safety and precision compared to freehand methods. A survey indicated that half-guided templates are more commonly used than full-guided ones in China. This study aims to assess the accuracy of implant placements using half-guided and full-guided digital surgical templates, considering factors like jaw location, tooth position, support type, implant timing, and bone density.

**Methods:**

87 implants (52 half-guided, 35 full-guided) were evaluated by comparing pre- and postoperative CBCT scans to measure deviations in coronal, apical, depth, and angular positions. Bone density was also assessed in relation to the implant deviations.

**Results:**

The findings revealed that the half-guided group exhibited significantly greater deviations in several areas: maxillary angular deviations, anterior coronal and depth deviations, posterior depth deviations, tooth-supported guide depth deviations, immediate implant coronal and angular deviations, and delayed implant depth deviations (*P* < 0.05). No significant differences were noted in other measurements. In the bone density analysis, only the full-guided group showed a significant negative correlation between bone density and apical deviation (*P* < 0.05).

**Conclusion:**

Based on statistical results, power calculations, and subgroup effect sizes, the following clinical recommendations are derived: Half-guided templates, owing to their superior cost-effectiveness in fabrication time and cost, are recommended for use in mandibular posterior regions, mucosa-supported templates, delayed implantations, and clinical scenarios with uneven bone density distribution at implant sites. In contrast, full-guided templates are more suitable for maxillary implantations, anterior regions, tooth-supported templates, immediate implantations, and sites with homogeneous bone density distribution.

## 1 Introduction

Before the widespread adoption of digitally guided implant technology, comprehensive clinical validation of its feasibility, safety, and accuracy is imperative. A fundamental clinical consideration remains the precise transfer of preoperative plans to the surgical site to ensure operational accuracy (1). This precision is influenced by cumulative errors throughout the treatment workflow, from data acquisition to surgical execution (2, 3). The process encompasses data collection, treatment planning, and guide fabrication utilizing CAD/CAM or 3D printing technologies (4, 5), with each stage potentially introducing deviations that may compromise final implant positioning (5).

Half-guided surgical templates assist in osteotomy preparation and sequential drilling. Still, they are removed before implant placement, whereas full-guided templates direct preparation and implant insertion, remaining *in situ* throughout the procedure. According to Chen et al. (6), half-guided templates are the predominant choice in China, with 88.1% of digital template users preferring this approach. Half-guided templates offer enhanced convenience, operational flexibility, and reduced production time and cost compared to full-guided systems. While some studies suggest superior accuracy with full-guided protocols (7–12), others report comparable precision between both techniques (13–16). Nevertheless, robust clinical evidence remains insufficient to establish the parity of half-guided templates in accuracy, and clinical selection criteria for different scenarios continue to present challenges.

Current literature primarily evaluates template accuracy within isolated cohorts or limited subgroups, constraining direct cross-protocol comparisons and impeding evidence-based clinical decision-making. To address this gap, our study analyzes clinical cases of 3D-printed half- and full-guided implant surgeries from Guangyuan Stomatological Hospital. We systematically evaluated both template types across multiple clinically relevant parameters—including jaw (maxilla/mandible), tooth position (anterior/posterior), support type (tooth-/mucosa-supported), implantation timing (immediate/delayed), and bone density. This comprehensive subgroup analysis facilitates rigorous head-to-head comparison and provides refined, clinically actionable guidance for template selection.

## 2 Methods

### 2.1 Case selection

Medical records and imaging data of patients who underwent oral implant surgery at Guangyuan Stomatological Hospital from April 2023 to March 2024 were collected. Patients were screened according to the following criteria:

#### 2.1.1 Criteria for inclusion

1. Aged 18 years or older with fully developed jawbones.
2. Missing teeth or requiring tooth extraction, with implant-supported fixed prosthetic restoration selection using a surgical guide.
3. Normal mouth opening capacity.
4. Fully informed of the implant treatment plan and voluntarily signed the informed consent form.
5. Availability of complete preoperative planning data and immediate postoperative CBCT records.

#### 2.1.2 Criteria for exclusion

1. Uncontrolled infections or inflammation at the implant site.
2. Uncontrolled systemic diseases or a history of jaw radiation therapy.
3. Restricted mouth opening capacity.
4. Specific oral mucosal diseases.
5. Severe bruxism or clenching habits.
6. Alcohol abuse, heavy smoking (>10 cigarettes/day), substance abuse, or drug addiction.
7. Missing or incomplete preoperative planning or postoperative CBCT data.

This study was conducted per the Declaration of Helsinki (revised in 2013) and was approved by the Medical Ethics Committee of Guangyuan Stomatological Hospital (Approval No.: GSHIRB-D-2023-301).

### 2.2 Data preparation

**Figure 1:**
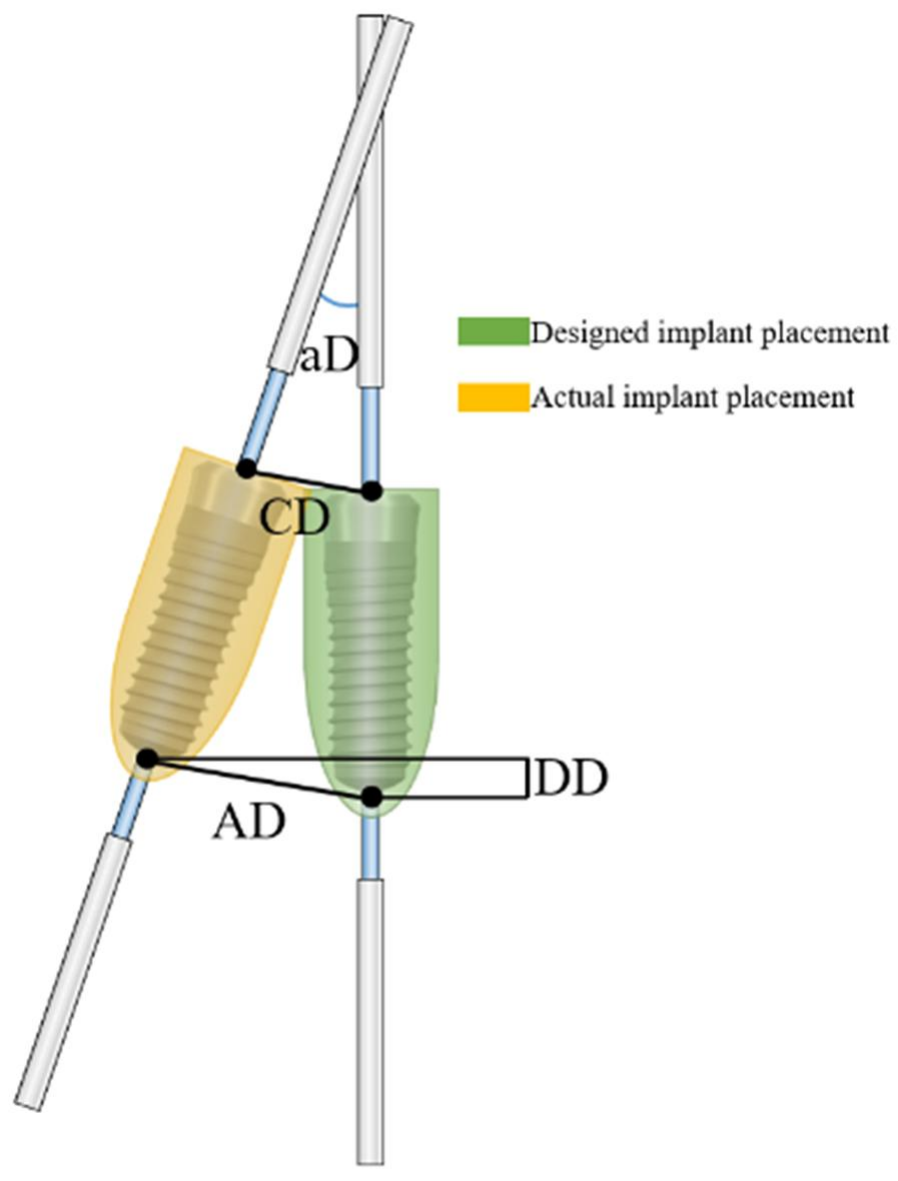
Diagram showing the deviations in features between the intended and final implant positions. (CD, coronal deviation; AD, apical deviation; DD, depth deviation; AD, angular deviation).

Implant positions were recorded using the Federation Dentaire Internationale (FDI) numbering system: 11–18, 21–28, 31–38, and 41–48. Implant accuracy was evaluated using four parameters: coronal deviation (CD), apical deviation (AD), angular deviation (aD), and depth deviation (DD), as illustrated in Figure 1. CD and AD represented the linear distances between the planned and actual implants' coronal and apical centers; aD was defined as the angle between their long axes; DD referred to the vertical distance between their apical centers.

**Figure 2:**
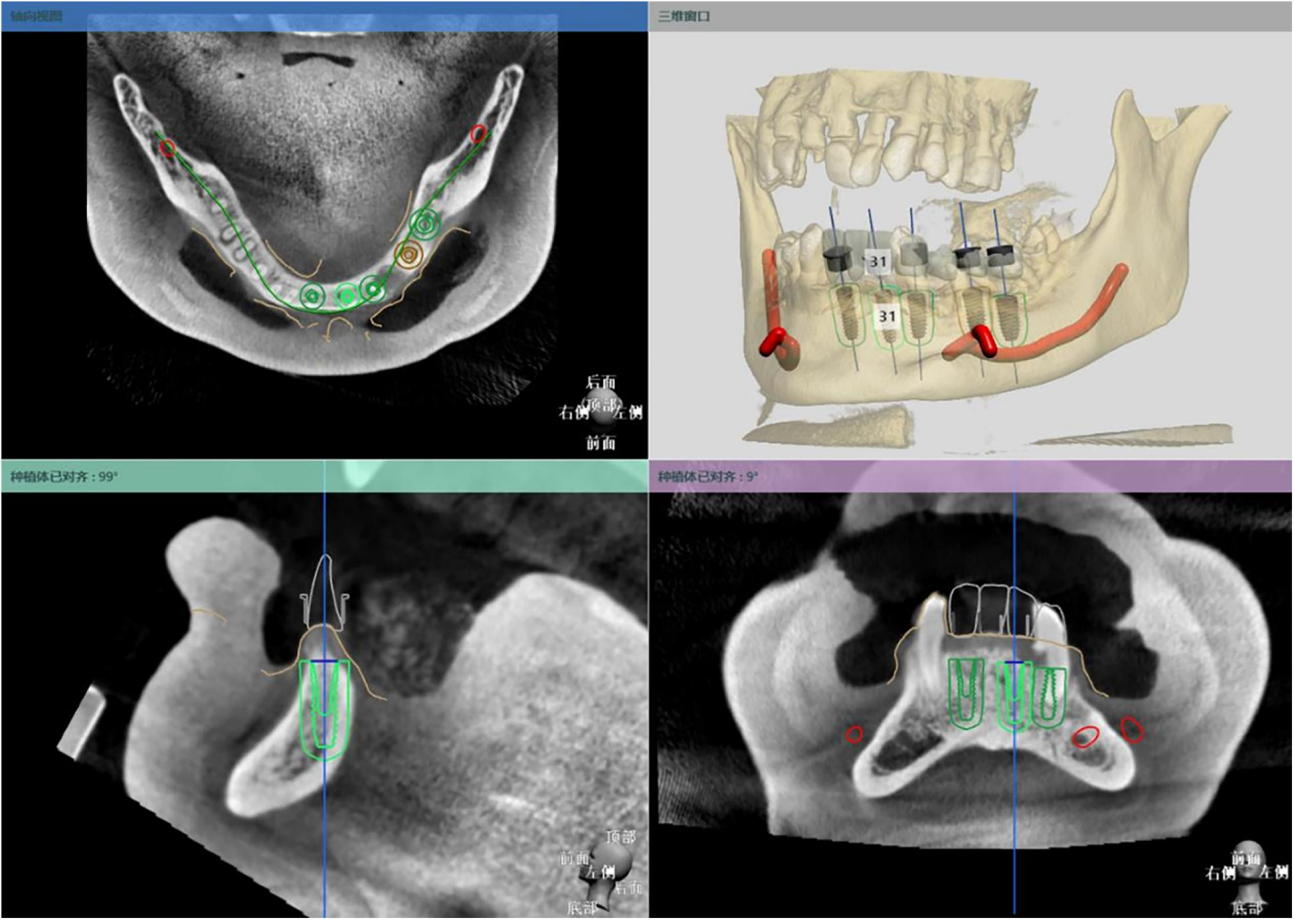
Virtual implant placement and treatment planning in 3Shape.

**Figure 3:**
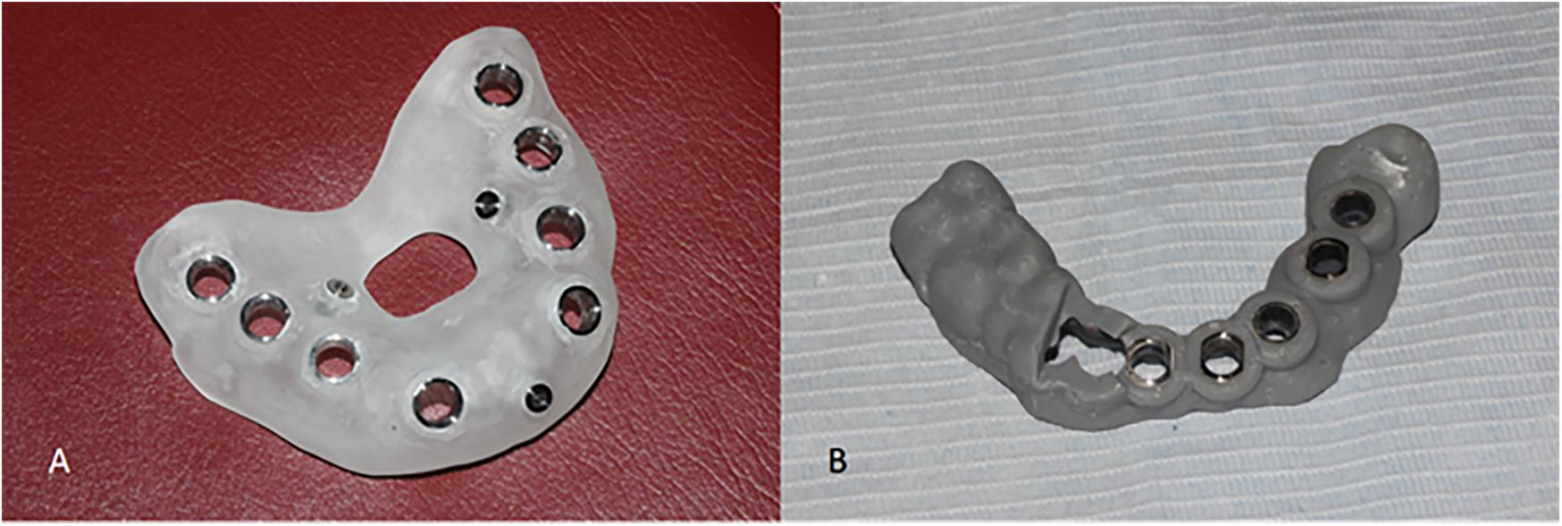
3d printed digital surgical templates. **(A)** Half-guided template. **(B)** Full-guided template.

All patients underwent pre- and postoperative CBCT scanning (HiRes3D-Plus, Langshi, China; 100 kV, 4 mA, 13 s, FOV: 16 cm × 10 cm), and data were saved in DICOM format. Preoperative intraoral scans were obtained using the PANDA P2 scanner (FREQTY, China). The CBCT and intraoral scan data were imported into 3Shape Dental System® (3Shape, Denmark) for image alignment and virtual implant planning, which was performed by an experienced technician and confirmed by the surgeon. Guide designs followed the restoration-driven principle, ensuring safe distances from adjacent anatomical structures (Figure 2). The guide models were exported in STL format, fabricated using SprintRay surgical resin (SP-RB0803) and a DLP 3D printer (SprintRay, USA), and then sterilized for clinical use. Representative half- and full-guided templates are shown in Figure 3 (**A,B**, respectively).

### 2.3 Surgical procedure

Under local infiltration anesthesia, surgical templates guided the incision, implant positioning, and socket preparation, including insufficient flap elevation when the attached gingiva was inadequate. For half-guided templates, implants were placed freehand after guided site preparation; for full-guided templates, implantation was fully guided. Dentium provided all surgical kits, implants, and sleeves. All procedures were performed by the same implant specialist with over 10 years of clinical experience, following a standardized digital protocol. The workflows for half- and full-guided surgeries are illustrated in Figuress 4, 5, respectively.

**Figure 4:**
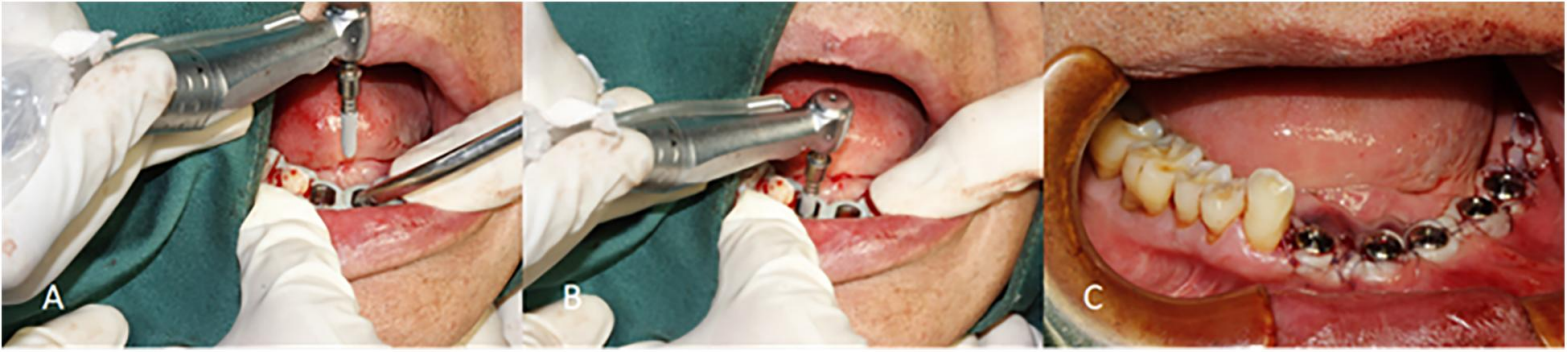
Surgery is performed using a half-guided template. **(A)** Osteotomy preparation using a drill and drill handle, guided by the half-guided surgical template. **(B)** Osteotomy site following the removal of the half-guided surgical template. **(C)** Implant placement performed freehand by the surgeon.

**Figure 5:**
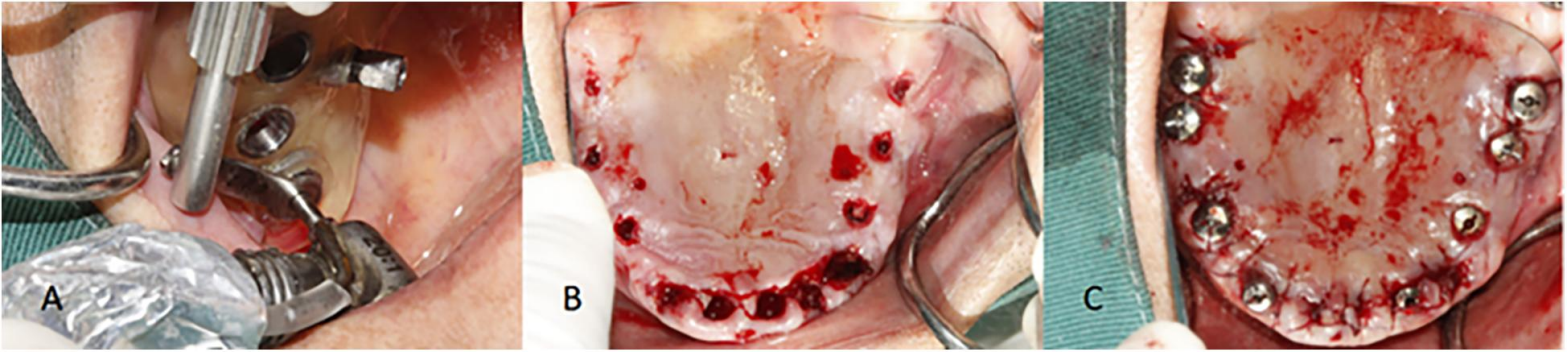
Surgery is performed using a full-guided template. **(A)** Implant placement preparation guided by the full-guided surgical template. **(B)** Implant depth control directed by the full-guided surgical template. **(C)** Implant placement into the prepared osteotomy site.

### 2.4 Accuracy measurement

Postoperative CBCT DICOM data were imported into Exocad for 3D reconstruction. CT thresholds were adjusted, and models were cropped to visualize the implants, adjacent teeth, and jawbone clearly. The STL files were imported into 3Shape and aligned with the preoperative design data. Section angles were adjusted to enable complete visualization and measurement of all parameters.

**Figure 6:**
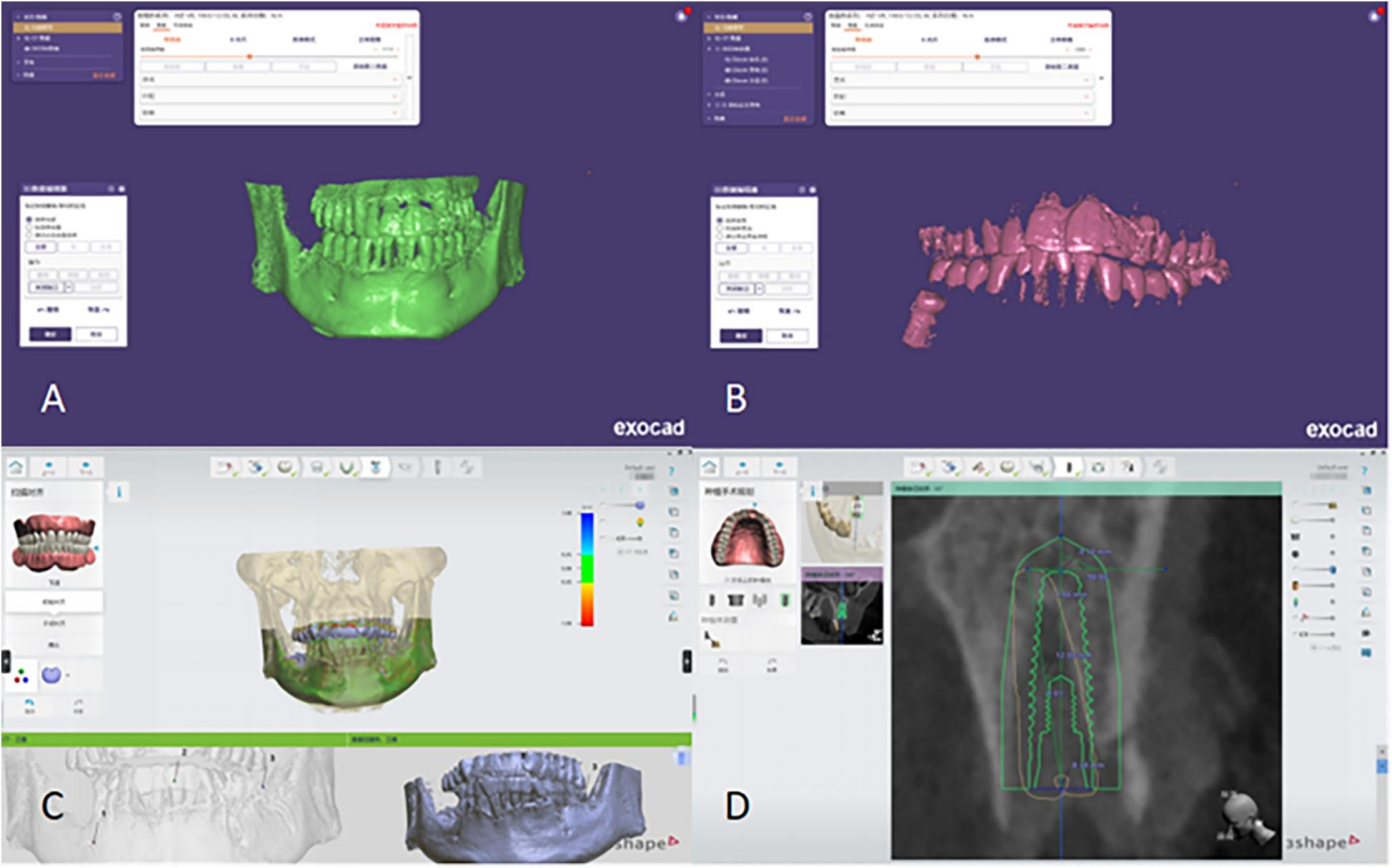
Conduct fitting and measurement of characteristic parameters post-surgery. **(A)** Extraction of the patient's jawbone STL file in Exocad. **(B)** Extraction of the patient's implant STL file in Exocad. **(C)** Import and alignment of the jawbone and implant STL files in 3Shape. **(D)** Measurement of characteristic deviations to assess implant accuracy.

​ To ensure measurement reliability, blinded assessments of all 87 implants were conducted independently by two Sichuan University and Guangyuan Stomatological Hospital examiners. Inter-examiner reliability was evaluated using intraclass correlation coefficients (ICC), with discrepancies exceeding 0.1 mm or 0.5° resolved through consensus. The detailed measurement workflow is illustrated in Figure 6.

### 2.5 Bone density measurement

**Figure 7:**
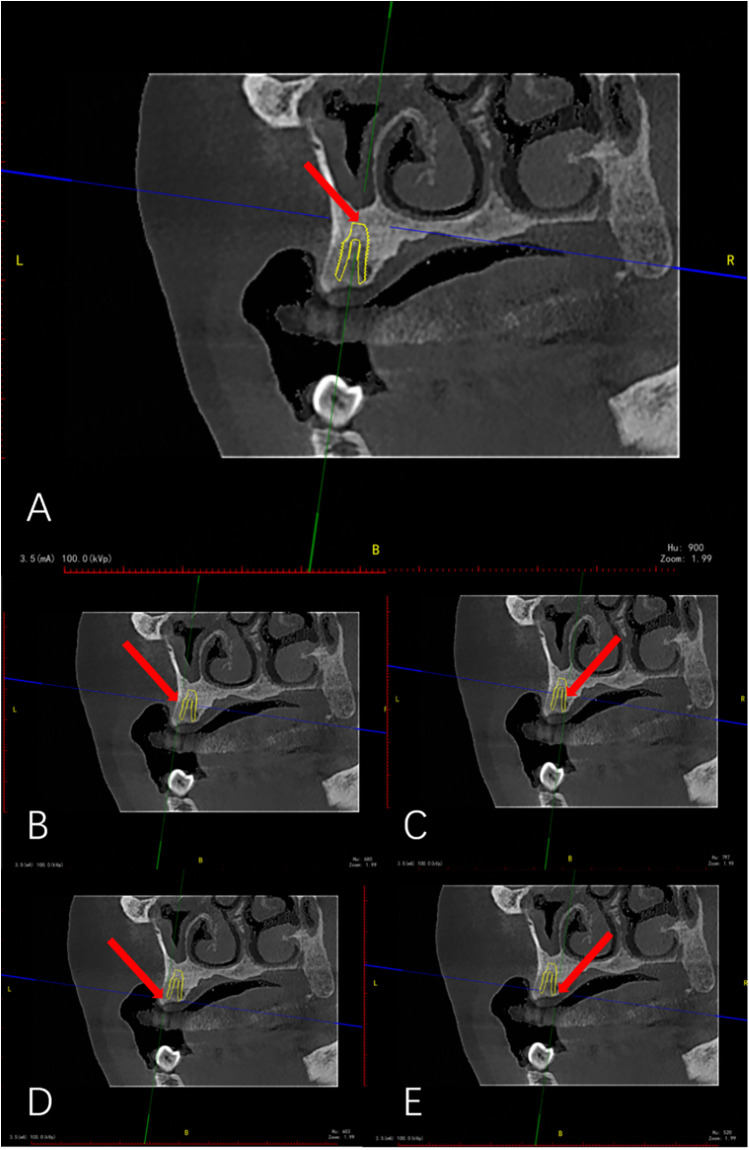
Bone densitometry (red arrow indicates bone densitometry point). **(A)** Bone density measurement (Apical). **(B)** Bone density measurement (Mid-buccal side). **(C)** Bone density measurement (Mid-palatal side). **(D)** Bone density measurement (Coronal buccal side). **(E)** Bone density measurement (Coronal palatal side).

In the coronal view of the preoperative CBCT image, bone density around the implant was measured. As shown in Figure 7, a green auxiliary line was drawn parallel to the implant's central axis, and a blue line was placed perpendicular to it. Their intersection points were positioned at the implant's apical, middle, and coronal regions. Bone density values were obtained at five locations: one at the apex, two at the middle (palatal and buccal sides), and two at the coronal region (palatal and buccal sides). The average of these five values was used for analysis. Through repeated assessments, two blinded examiners independently performed bone density measurements (one from Sichuan University and one from Guangyuan Stomatological Hospital). The intraclass correlation coefficient (ICC) was calculated to evaluate interobserver reliability.

### 2.6 Grouping of factors affecting accuracy

1. Jaw (Maxilla vs. Mandible): Patients were grouped based on whether the implant was placed in the maxilla or mandible.
2. Tooth Position (Anterior vs. Posterior): Patients were categorized according to implant location in the anterior (FDI 11–13, 21–23, 31–33, 41–43) or posterior regions (FDI 14–18, 24–28, 34–38, 44–48).
3. Template Support Type (Tooth-Supported vs. Mucosa-Supported): Patients were classified based on whether the surgical guide was tooth-supported or mucosa-supported.
4. Implantation Timing (Immediate vs. Delayed): Patients were divided by implantation timing into immediate (post-extraction) or delayed (16–20 weeks post-extraction) groups.

### 2.7 Statistical analysis

All data were analyzed using descriptive statistics, including mean and standard deviation (SD). Independent *t*-tests or Mann–Whitney *U* tests were performed for grouped data depending on the normality of distribution, and significance was determined based on the corresponding test results. Pearson correlation analysis was conducted between bone density and characteristic parameters, with statistical significance set at *P* < 0.05. All analyses were performed using SPSS software (version 29.0; SPSS Inc., Chicago, IL, USA).

A *post-hoc* power calculation was performed to evaluate the statistical power of this study and mitigate the risk of a Type II error, accompanied by a corresponding effect size analysis.

## 3 Results

The types, characteristics, and bone density of the 87 implants used at Guangyuan Stomatological Hospital are presented in Supplementary Table S1. The characteristic deviations observed in oral implant surgery guided by full-guided (35 implants) and half-guided (52 implants) digital surgical templates are summarized in Table 1. Interobserver reliability, assessed via blinded measurements of characteristic parameters by two independent examiners, yielded excellent intraclass correlation coefficients (ICC): 0.91 for coronal deviation, 0.87 for apical deviation, 0.89 for angular deviation, 0.90 for depth deviation, and 0.86 for bone density.

**Table 1:** Characteristic deviations in implant accuracy between half-guides and full-guides.

Template type	Coronal deviation		Apical deviation		Angular deviation		Depth deviation	
	Mean ± SD (mm)	*P*-Value	Mean ± SD (mm)	*P*-Value	Mean ± SD (°)	*P*-Value	Mean ± SD (mm)	*P*-Value
Full-guided template	0.96 (±0.51)	0.040*	1.14 (±0.6)	0.248	3.15 (±2.17)	0.017*	0.78 (±0.44)	0.003**
Half-guided template	1.2 (±0.53)		1.32 (±0.74)		5.27 (±5.68)		1.13 (±0.62)	

* *P* < 0.05.

** *P* < 0.01.

**Figure 8:**
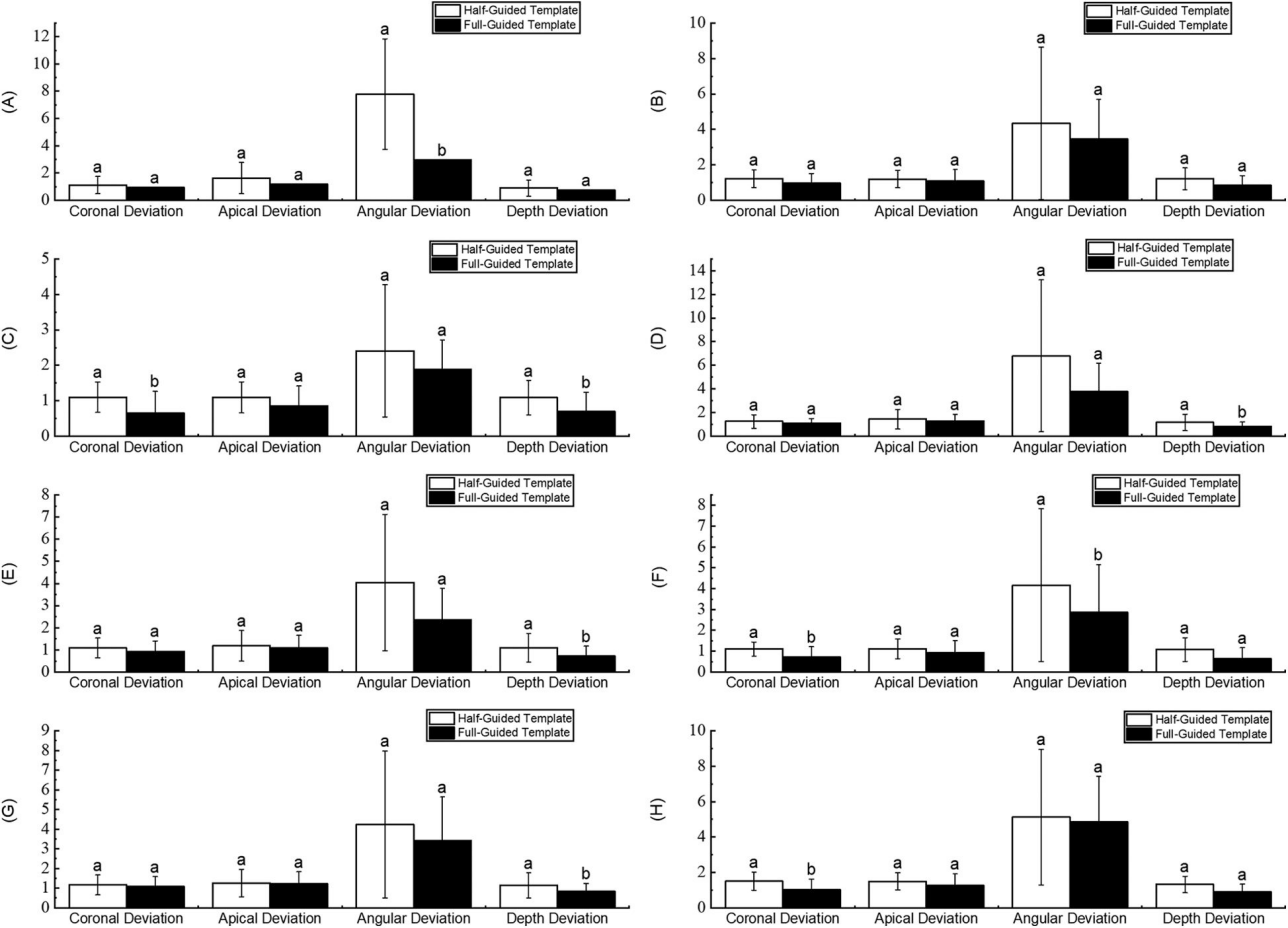
Comparison of characteristic parameters in different groups. **(A)** Maxilla. **(B)** Mandible. **(C)** Anterior teeth. **(D)** Posterior teeth. **(E)** Tooth-supported. **(F)** Mucosa-supported. **(G)** Immediate implant. **(H)** Delayed implant.

As shown in Table 1, statistically significant differences (*P* < 0.05) were observed between the half-guided and full-guided surgical templates regarding overall characteristic deviations, including coronal deviation, angular deviation, and depth deviation. However, no significant difference was found in apical deviation. A more detailed analysis of these characteristic parameters for full-guided and half-guided surgical templates across various groups can be found in Supplementary Table S2. The results of this analysis are illustrated in Figure 8.

In Figure 8, it is evident that in the jaw group (comparing the maxilla and mandible), the half-guided and full-guided surgical templates showed statistically significant differences only in the angular deviation of the maxilla, with mean deviations of 1.12 ± 0.63 and 0.95 ± 0.50, respectively (*P* = 0.045). No other accuracy deviations within this group were statistically significant. In the tooth position group (comparing anterior and posterior regions), half-guided and full-guided templates showed substantial differences in coronal and depth deviations in the anterior region (*P* = 0.029 and 0.046, respectively), as well as in depth deviation in the posterior region (*P* = 0.044). In the template support type group (tooth-supported vs. mucosa-supported), statistically significant differences were observed only in-depth deviation within the tooth-supported subgroup, with mean deviations of 1.09 ± 0.65 and 0.73 ± 0.44 (*P* = 0.016). In contrast, other deviations were not significantly different. In the implantation timing group (immediate vs. delayed), half-guided and full-guided templates exhibited significant differences in coronal and angular deviations in the immediate implantation subgroup (*P* = 0.01 and 0.021, respectively), and in-depth deviation in the delayed implantation subgroup (*P* = 0.039).

Table 2 shows the correlation between the characteristic parameters of implants guided by full-guided and half-guided surgical templates and bone density. As shown in Table 2, apical deviation in the full-guided surgical template group was significantly negatively correlated with bone density (*r* = −0.351*, *P* < 0.05). However, no significant correlations were found between bone density and coronal deviation (*r* = −0.148), angular deviation (*r* = −0.296), or depth deviation (*r* = 0.130) in the full-guided group (*P* > 0.05). In the half-guided surgical template group, no significant correlations were observed between bone density and any of the characteristic deviations, including coronal deviation (*r* = −0.055), apical deviation (*r* = −0.138), angular deviation (*r* = 0.005), or depth deviation (*r* = 0.163), under the conditions of the present study (*P* > 0.05).

**Table 2:** Correlation between the characteristic parameters of implants using full-guided and half-guided surgical templates and bone density.

Factors affecting accuracy	Template type	Coronal deviation	Apical deviation	Angular deviation	Depth deviation
Bone density	Full-guided	−0.148	−0.351*	−0.296	0.130
	Half-guided	−0.055	−0.138	0.005	0.163
Coronal deviation	Full-guided	1	0.541**	0.392*	0.422*
	Half-guided	1	0.525**	0.223	0.471**
Apical deviation	Full-guided		1	0.430*	0.394*
	Half-guided		1	0.668**	0.223
Angular deviation	Full-guided			1	0.194
	Half-guided			1	0.123
Depth deviation	Full-guided				1
	Half-guided				1

* *P* < 0.05.

** *P* < 0.01.

The *post-hoc* power calculation conducted in this study indicated sufficient statistical power (≥ 0.70) for most comparisons, demonstrating a robust ability to detect clinically meaningful differences. Notably, the comparison for depth deviation yielded a power of 0.837, providing strong evidence. Conversely, lower power was observed in specific small-sample subgroups, such as for angular deviation in the immediate implantation group (power = 0.15), and for apical deviation overall (power = 0.227). The results from these underpowered comparisons should therefore be interpreted with caution. Detailed results of the power calculation are presented in Supplementary Table S3.

Supported by *post-hoc* power and effect size calculations (see Supplementary Table S3), this study provides more substantial evidence to inform the selection of half-guided and full-guided templates across diverse clinical scenarios. In specific comparisons, such as for depth and coronal deviations, the full-guided protocol demonstrated medium-to-large effect sizes (e.g., Cohen's *d* = 0.651 for depth deviation), offering substantial clinical evidence of its utility, particularly in maxillary and immediate implantation cases (Cohen's *d* = 1.525, indicating a significant effect). Conversely, the half-guided template also showed considerable effectiveness in other situations, such as coronal deviation in the anterior region (Cohen's *d* = 0.794, a medium-to-large effect).

## 4 Discussion

### 4.1 Precision in digital guidance for implant surgery

Computer-assisted implantation using surgical guides enables more accurate implant placement and offers advantages such as high efficiency, reduced pain, and minimal bone loss (17). However, its accuracy remains controversial, influenced by factors including support type (tooth- vs. mucosa-supported), guide type (half- vs. full-guided), and operator experience (18–20). Unlike half-guided templates, which assist only with positioning and allow freehand implant placement, full-guided templates control the entire procedure. Despite higher costs and limitations related to interocclusal space (21), several studies have demonstrated that full-guided templates provide superior accuracy (7–11, 22), with some proposing them as the gold standard (12). Nevertheless, other studies have reported comparable accuracy between half- and full-guided templates (13–16). This study compared implant accuracy between half- and full-guided templates at Guangyuan Stomatological Hospital to provide evidence for clinical decision-making.

### 4.2 Comparison of implant accuracy with international digital template studies

A recent meta-analysis on fully guided implant placement accuracy reported mean deviations of 1.1–1.4 mm for coronal deviation, 1.2–1.6 mm for apical deviation, 3.0–3.8° for angular deviation, and 0.46 mm for depth deviation, as summarized in Table 3 (16, 22–25). The deviations observed in this study for fully guided templates at Guangyuan Stomatological Hospital were consistent with these international standards. In comparison, half-guided templates showed slightly larger deviations, although the overall trend aligned with findings from multiple meta-analyses.

**Table 3:** Comparison of data from international studies and this study.

Data source	Year of study	Number of implants	Coronal deviation/mm	Apical deviation/mm	Angular deviation/°	Depth deviation/mm
Van Assche et al. (16)	2012	1,688	1.09	1.28	3.81	0.46
Tahmaseb et al. (23)	2018	2,238	1.2	1.4	3.5	/
López et al. (24)	2019	2,767	1.14	1.46	3.08	/
Lin et al. (22)	2020	43	0.57 (±0.33)	1.14 (±0.72)	4.30 (±2.87)	0.46 (±0.36)
Tresserra et al. (25)	2021	/	1.4 (±0.7)	1.6 (±0.7)	3.0 (±2.0)	/
Guangyuan Stomatological Hospital full-guided template	2023	35	0.96 (±0.51)	1.14 (±0.6)	3.15 (±2.17)	0.78 (±0.44)
Guangyuan Stomatological Hospital half-guided template	2023	52	1.2 (±0.53)	1.32 (±0.74)	5.27 (±5.68)	1.13 (±0.62)

### 4.3 Analysis of implant accuracy in half-guided and full-guided templates

#### 4.3.1 Maxilla and mandible

No significant differences were found between half-guided and full-guided templates in the maxilla's coronal, apical, or depth deviations. In contrast, angular deviation showed marginal significance (*P* = 0.045), possibly due to the small sample size. In the mandible, none of the four deviations differed significantly between template types. Overall, implant accuracy was comparable for both jaws across template groups. Although a slight difference in angular deviation was observed in the maxilla, clinical measures such as personalized abutments and occlusal force adjustments can effectively mitigate its impact. No patient discomfort was reported during follow-up, indicating satisfactory accuracy of half-guided templates in the upper jaw. Therefore, full-guided templates are recommended for maxillary implants, while half-guided templates are appropriate for mandibular cases.

#### 4.3.2 Anterior vs. posterior teeth

Comparison of deviation values between half-guided and full-guided surgical templates in anterior and posterior tooth regions revealed notable findings. In the anterior region, the coronal deviation of half-guided templates was slightly greater than that of full-guided templates, with a statistically significant difference (*P* = 0.029). However, apical and angular deviations did not differ significantly. Depth deviation showed a statistical difference (*P* = 0.046), but considering the potential influence of sample size and the overall non-significant differences, its clinical relevance requires further evaluation. These results indicate that while full-guided templates provide superior coronal control anteriorly, half-guided templates perform comparably in apical and angular deviations. In the posterior region, half-guided templates exhibited slightly greater deviations in coronal, apical, and angular parameters than full-guided templates, with statistical significance only observed in depth deviation (*P* = 0.044). Given the aesthetic importance of the anterior region, it is recommended that clinicians prioritize full-guided templates or use half-guided templates with caution in this area. Half-guided templates may be appropriately selected in the posterior region, where only depth deviation differed significantly.

#### 4.3.3 Template support type

Statistical results from Guangyuan Stomatological Hospital showed that tooth-supported templates achieved better accuracy than mucosa-supported templates across all four deviation parameters, regardless of whether half-guided or full-guided surgical templates were used. Although tooth-supported half-guided templates exhibited slightly greater deviations than full-guided templates in all characteristics, the difference was significant only in the depth deviation (*P* = 0.016), suggesting an advantage of tooth-supported full-guided templates in controlling implant depth. No significant differences were found between half-guided and full-guided templates in coronal, apical, and angular deviations, indicating that both provide stable support and ensure accurate implant angulation. No significant differences were observed between the two template types for any deviation parameter in the mucosa-supported group. These findings align with a systematic review and meta-analysis reporting that tooth-supported guides are more accurate than mucosa-supported ones (26). Mucosa-supported templates tend to exhibit greater deviations overall. To enhance the accuracy of mucosa-supported guides, Mai et al. (27) suggested that rigid support at the edentulous end, such as using micro-screws as substitutes for teeth, effectively reduces template movement and increases stability. Furthermore, Chen et al.'s (6) questionnaire survey ranked template support type as a significant factor influencing implant accuracy, underscoring the importance of considering this variable. Therefore, full-guided templates are recommended for tooth-supported surgeries, while half-guided templates may be preferable for mucosa-supported cases.

### 4.3.4 Implant timing

Immediate implantation refers to implant placement immediately after tooth extraction, whereas delayed implantation is performed following a healing period. During immediate implantation, the lingual bone wall exhibits greater resistance than the buccal side, causing the drill to deviate toward the buccal side—a factor that may compromise implantation accuracy. Although surgical templates aid in more precise implant positioning, drill slippage can still occur during osteotomy preparation. Our statistical results demonstrated that half-guided templates exhibited larger deviations than full-guided ones in immediate and delayed implantation. Specifically, coronal deviation (*P* = 0.01) and angular deviation (*P* = 0.021) in immediate implantation, as well as depth deviation (*P* = 0.039) in delayed implantation, reached statistical significance. These findings suggest that half-guided templates may demonstrate inferior accuracy compared to full-guided templates in immediate implantation. Consistent with our results, Chen et al. (9) reported that full-guided templates outperformed half-guided templates in angular and depth deviations during immediate implantation. In contrast, full-guided templates showed superiority in depth deviation in delayed implantation. Therefore, we recommend half-guided templates for delayed implantation procedures and full-guided templates for immediate implantation.

### 4.4 Bone density

Previous studies have reported correlations between angular deviation and bone density (20, 28). In the present study, bone density measurements (in Hounsfield Units) obtained from five predetermined sites around the implant socket on preoperative CBCT scans revealed a statistically significant negative correlation with apical deviation (*r* = –0.351, *P* < 0.05) and a weak negative correlation with angular deviation (*r* = –0.296) in the full-guided group, a trend similarly observed in the half-guided group.

Bone density exhibits considerable heterogeneity across anatomical regions, including between the maxilla and mandible, anterior and posterior areas, and even at different sites of the same tooth. Owing to the path-of-least-resistance principle, drill bits tend to deviate from the planned trajectory in low-density bone, which can lead to angular or positional implant inaccuracies. Under these conditions, half-guided templates provide greater operative flexibility, enabling surgeons to make real-time adjustments—such as reducing insertion force in low-density areas or avoiding excessive pressure in dense bone—based on tactile feedback. However, this flexibility introduces the risk of operator-dependent variability. In contrast, while the rigid design of full-guided systems ensures higher precision, it offers limited intraoperative adaptability when actual bone density differs from preoperative assessments, potentially resulting in suboptimal implant positioning.

Therefore, for patients with relatively homogeneous bone density, full-guided protocols are recommended to maximize stability and accuracy by minimizing procedural deviations. Conversely, in cases with significant bone density heterogeneity, half-guided templates are advantageous due to their superior operative flexibility, particularly when managing low or uneven bone density.

Furthermore, the choice of guide type should be informed by specific anatomical challenges. The maxilla, typically exhibiting lower bone density (often ≥300 HU) and frequently presenting with reduced bone volume and proximity to the maxillary sinus in the posterior region, benefits from the precise trajectory control of full-guided templates. This approach helps prevent sinus perforation and is particularly indicated in complex cases requiring bone augmentation, such as sinus floor elevation. In contrast, the mandible, despite its higher density (usually ≥500 HU), houses the mandibular canal, demanding stringent surgical accuracy. Here, a half-guided protocol, which provides initial guidance for the pilot drill while allowing subsequent tactile adjustments, can effectively balance procedural efficiency with the mitigation of nerve injury risk. For cases with markedly abnormal or heterogeneous bone density that may exceed the compensatory capacity of conventional guides, the utilization of dynamic navigation or customized templates with augmented support structures should be considered as a viable strategy to further mitigate deviation risks.

### 4.5 The impact of operator experience on implantation accuracy

We recognize that operator experience may influence the accuracy of guided surgery, which constitutes an important factor worthy of consideration. To better understand its impact, we previously conducted a nationwide survey specifically designed to evaluate the relative influence of 16 factors, including operator experience, on the accuracy of digitally guided implant placement (6). The results indicated that operator experience was ranked 8th among the factors in terms of its perceived impact. This finding indeed suggests differences in implantation accuracy between novice and experienced operators when using digital guides. However, to standardize experimental conditions and minimize confounding variables, the present study deliberately utilized data from a single experienced implant surgeon at one hospital. This approach was adopted to ensure data reliability and to provide recommendations for selecting guide types under different clinical scenarios. Currently, our research group is collecting multi-center data on implantation accuracy from both novice and experienced operators, which will allow for a more robust evaluation of the effect of operator experience on the accuracy of digitally guided implant placement.

### 4.6 Limitations of this study

Evaluating the accuracy of half-guided and full-guided surgical templates is meaningful, providing clinical suggestions for digitally guided implant surgery. However, this study was conducted retrospectively, possibly introducing selection and information biases. All cases were collected from a single center and included only those with complete datasets, potentially limiting the sample's representativeness. Additionally, bone density measurements were not normalized, relying solely on power calculations and effect sizes for analytical support. Variability in imaging and measurement procedures may also affect accuracy. To mitigate bias, standardized protocols and independent double measurements were employed. Nonetheless, these inherent limitations restrict causal inference and the generalizability of the findings. Future large-scale prospective randomized studies are needed to validate these results.

## 5 Conclusions

This study compared implant deviations between half- and full-guided surgical templates by evaluating predefined subgroups (jaw, tooth position, support type, implantation timing, and bone density). Within the study limitations, the analysis of effect sizes and statistical power demonstrates that half-guided templates are a clinically adequate and cost-effective option for mandibular, posterior, mucosa-supported, and delayed implant placements, particularly in cases with heterogeneous bone density distribution. Conversely, full-guided templates are indispensable in scenarios requiring high precision, such as maxillary, anterior, tooth-supported, and immediate implantations, especially when bone density is relatively uniform. These findings highlight the clinical importance of selecting the appropriate guide type based on specific conditions. Future research should involve prospective, multicenter clinical trials to further validate the accuracy of digitally guided implant placement and promote its standardized clinical implementation.

## Data Availability

The original contributions presented in the study are included in the article/Supplementary Material, further inquiries can be directed to the corresponding author.

## References

[1] Li J Ou G. Accuracy of computer-guided implant placement and influencing factors. West China J Stomatol. (2017) 35:93–8. 10.7518/hxkq.2017.01.015 PMC7030204 28326735

[2] Miller RJ Bier J. Surgical navigation in oral implantology. Implant Dent. (2006) 15:41–7. 10.1097/01.id.0000202637.61180.2b 16569960

[3] Schneider D Marquardt P Zwahlen M Jung RE. A systematic review on the accuracy of computer-guided template-based implant dentistry. Clin Oral Implants Res. (2009) 20(Suppl 4):73–86. 10.1111/j.1600-0501.2009.01788.x 19663953

[4] Xu L You J Zhang J Liu Y Peng W. Impact of surgical template on implant placement accuracy. J Prosthodont. (2016) 25:641–6. 10.1111/jopr.12407 26619380

[5] Valente F Schiroli G Sbrenna A. Accuracy of computer-aided oral implant surgery. Int J Oral Maxillofac Implants. (2009) 24:234–42. 19492638

[6] Chen Y Su B. Application of digital guide templates in China. BMC Oral Health. (2023) 23:36. 36683029 10.1186/s12903-023-02750-4 PMC9869612

[7] Varga E Jr Antal M Major L Kiscsatári R Braunitzer G Piffkó J. Freehand vs. guided dental implantation. Clin Oral Implants Res. (2020) 31:417–30. 10.1111/clr.13578 31958166

[8] Lou F Rao P Zhang M Luo S Lu S Xiao J. Accuracy evaluation of guided templates in anterior teeth. Clin Implant Dent Relat Res. (2021) 23:117–30. 10.1111/cid.12980 33528110

[9] Chen Y Zhang X Wang M Jiang Q Mo A. Accuracy of guided templates in anterior implantation. Materials (Basel). (2020) 14:26. 10.3390/ma14010026 33374727 PMC7793484

[10] Schulz MC Hofmann F Range U Lauer G Haim D. Guided vs. full-guided implant insertion. Int J Implant Dent. (2019) 5:23. 31240421 10.1186/s40729-019-0176-4 PMC6593025

[11] Søndergaard K Hosseini M Storgård Jensen S Spin-Neto R Gotfredsen K. Guided vs. conventional implant placement. Clin Oral Implants Res. (2021) 32:1072–84. 10.1111/clr.13802 34166539

[12] Younes F Cosyn J De Bruyckere T Cleymaet R Bouckaert E Eghbali A. Accuracy of guided implant surgery. J Clin Periodontol. (2018) 45:721–32. 10.1111/jcpe.12897 29608793

[13] Sarhan MM Khamis MM El-Sharkawy AM. Accuracy of full-guided versus half-guided template-assisted implant placement: a split-mouth clinical study. J Prosthet Dent. (2021) 125:620–7. 10.1016/j.prosdent.2020.02.025 32389377

[14] Kraft B Frizzera F de Freitas RM de Oliveira GJLP Marcantonio Junior E. Comparison of full-guided and pilot-drill guided implant surgery: a randomized controlled clinical trial. Clin Implant Dent Relat Res. (2020) 22:631–7. 10.1111/cid.12941 32875722

[15] Kim HJ Kim HJ Moon SY. Comparison of the accuracy of dental implant placement between full-guided and pilot-guided surgery systems in different support types. Appl Sci. (2020) 10:1975. 10.3390/app10061975

[16] Van Assche N Vercruyssen M Coucke W Teughels W Jacobs R Quirynen M. Accuracy of computer-aided implant placement. Clin Oral Implants Res. (2012) 23(Suppl 6):112–23. 10.1111/j.1600-0501.2012.02552.x 23062136

[17] Tallarico M Park C-J Lumbau AI Annucci M Baldoni E Koshovari A Customized 3D-printed titanium mesh. Materials (Basel). (2020) 13:3874. 10.3390/ma13173874 32887390 PMC7503418

[18] Hämmerle CH Cordaro L van Assche N Benic GI Bornstein M Gamper F Digital technologies in implant dentistry. Clin Oral Implants Res. (2015) 26(Suppl 11):97–101. 26385624 10.1111/clr.12648

[19] Spielau T Hauschild U Katsoulis J. Template-guided immediate implant placement. BMC Oral Health. (2019) 19:55. 10.1186/s12903-019-0746-0 30975113 PMC6460533

[20] Cassetta M Di Mambro A Giansanti M Stefanelli LV Cavallini C. Error of stereolithographic surgical templates. Int J Oral Maxillofac Surg. (2013) 42:264–75. 10.1016/j.ijom.2012.06.010 22789635

[21] Kühl S Zürcher S Mahid T Müller-Gerbl M Filippi A Cattin P. Accuracy of guided implant surgery. Clin Oral Implants Res. (2013) 24:763–9. 10.1111/j.1600-0501.2012.02484.x 22551385

[22] Lin C-C Wu C-Z Huang M-S Huang C-F Cheng H-C Wang DP. Fully digital workflow for guided surgery. J Clin Med. (2020) 9:980. 10.3390/jcm9040980 32244735 PMC7231012

[23] Tahmaseb A Wu V Wismeijer D Coucke W Evans C. Accuracy of static computer-aided surgery. Clin Oral Implants Res. (2018) 29(S16):416–35. 10.1111/clr.13346 30328191

[24] Sigcho López DA García I Da Silva Salomao G Cruz Laganá D. Deviation factors in surgical guides. Implant Dent. (2019) 28:68–73. 10.1097/ID.0000000000000853 30640309

[25] Parra-Tresserra A Marquès-Guasch J Ortega-Martínez J Basilio-Monné J Hernández-Alfaro F. Dynamic surgery review. Med Oral Patol Oral Cir Bucal. (2021) 26:e576–81. 10.4317/medoral.24566 34023841 PMC8412455

[26] Raico Gallardo YN da Silva-Olivio IR Mukai E Morimoto S Sesma N Cordaro L. Accuracy comparison of guided surgery. Clin Oral Implants Res. (2017) 28:602–12. 27062555 10.1111/clr.12841

[27] Mai HN Lee DH. Stabilization of implant guide templates. J Prosthet Dent. (2020) 124:727.e1–8. 33160620 10.1016/j.prosdent.2020.06.033

[28] Ozan O Orhan K Turkyilmaz I. Correlation between bone density and implant deviation. J Craniofac Surg. (2011) 22:1755–61. 10.1097/SCS.0b013e31822e6305 21959426

