# Supplementary material for: Assessing the precision of 3D-printed surgical templates in oral implant placement: a comparison of half and full-guided approaches
Source: Front Dent Med. 2025 Nov 26;6:1700363. doi: 10.3389/fdmed.2025.1700363 (PMC12689983; doi:10.3389/fdmed.2025.1700363)
Supplement: Supplementary file 1 [file Table1.doc]

**Table S1**

Guide plate types, characteristic parameters, and bone density of the 87 dental implants used at Guangyuan Dental Hospital

| Implant No. | tooth position | Template Restriction Type | Jaw | Coronal Deviation（mm） | Apical Deviation（mm） | Angular Deviation（°） | Depth Deviation（mm） | Implant Timing | Bone Density（HU） |
| --- | --- | --- | --- | --- | --- | --- | --- | --- | --- |
| 1 | 35 | Half-guided | Mandible | 1.32 | 1.22 | 5.01 | 1.18 | Delayed Implant | 1120 |
| 2 | 37 | Half-guided | Mandible | 1.79 | 1.33 | 0.06 | 1.32 | Delayed Implant | 890 |
| 3 | 45 | Half-guided | Mandible | 1.04 | 1.1 | 7.23 | 1.12 | Delayed Implant | 1035 |
| 4 | 47 | Half-guided | Mandible | 1.05 | 1.23 | 1.39 | 1.86 | Delayed Implant | 850 |
| 5 | 26 | Half-guided | Maxilla | 0.29 | 0.61 | 2.85 | 0.59 | Delayed Implant | 620 |
| 6 | 27 | Half-guided | Maxilla | 1.2 | 1.21 | 0.85 | 0.2 | Delayed Implant | 570 |
| 7 | 42 | Half-guided | Mandible | 1.91 | 0.94 | 5.48 | 1.92 | Delayed Implant | 1100 |
| 8 | 31 | Half-guided | Mandible | 1.09 | 1.64 | 1.3 | 0.91 | Immediate Implant | 1020 |
| 9 | 26 | Half-guided | Maxilla | 1.06 | 1.44 | 3.03 | 0.46 | Delayed Implant | 680 |
| 10 | 27 | Half-guided | Maxilla | 0.07 | 0.46 | 3.72 | 0.54 | Delayed Implant | 740 |
| 11 | 25 | Half-guided | Maxilla | 0.96 | 1.17 | 1.43 | 1.09 | Delayed Implant | 750 |
| 12 | 27 | Half-guided | Maxilla | 1.24 | 0.6 | 5.62 | 0.59 | Delayed Implant | 650 |
| 13 | 31 | Half-guided | Mandible | 0.95 | 1.25 | 2.65 | 1.58 | Delayed Implant | 820 |
| 14 | 33 | Half-guided | Mandible | 0.4 | 0.44 | 1.54 | 0.39 | Delayed Implant | 1000 |
| 15 | 37 | Half-guided | Mandible | 0.11 | 0.16 | 0.61 | 0.11 | Delayed Implant | 500 |
| 16 | 41 | Half-guided | Mandible | 1.57 | 1.6 | 5.33 | 1.58 | Delayed Implant | 620 |
| 17 | 43 | Half-guided | Mandible | 1.66 | 1.65 | 1.37 | 1.65 | Delayed Implant | 550 |
| 18 | 45 | Half-guided | Mandible | 0.87 | 0.74 | 4.77 | 0.69 | Delayed Implant | 1150 |
| 19 | 16 | Half-guided | Maxilla | 1.72 | 1.79 | 8.02 | 1.12 | Delayed Implant | 732 |
| 20 | 17 | Half-guided | Maxilla | 0.96 | 1.86 | 2.01 | 0.75 | Delayed Implant | 620 |
| 21 | 26 | Half-guided | Maxilla | 0.76 | 0.41 | 2.73 | 0.96 | Delayed Implant | 570 |
| 22 | 27 | Half-guided | Maxilla | 1.27 | 1.72 | 1.42 | 1.33 | Delayed Implant | 618 |
| 23 | 45 | Half-guided | Mandible | 1.14 | 2.21 | 16.01 | 2.8 | Delayed Implant | 980 |
| 24 | 47 | Half-guided | Mandible | 1.93 | 1.81 | 17.2 | 1.9 | Delayed Implant | 860 |
| 25 | 32 | Half-guided | Mandible | 0.51 | 0.39 | 0.39 | 0.31 | Immediate Implant | 1100 |
| 26 | 42 | Half-guided | Mandible | 1.29 | 1.3 | 1.1 | 1.03 | Immediate Implant | 950 |
| 27 | 11 | Half-guided | Maxilla | 0.76 | 1.43 | 5.81 | 1.68 | Delayed Implant | 950 |
| 28 | 21 | Half-guided | Maxilla | 0.89 | 0.52 | 2.22 | 0.89 | Immediate Implant | 860 |
| 29 | 24 | Half-guided | Maxilla | 1.29 | 1.23 | 13.11 | 2.36 | Immediate Implant | 1000 |
| 30 | 26 | Half-guided | Maxilla | 1.22 | 4.4 | 2.17 | 0.17 | Delayed Implant | 850 |
| 31 | 35 | Half-guided | Mandible | 1.4 | 1.42 | 5.85 | 1.85 | Delayed Implant | 1100 |
| 32 | 36 | Half-guided | Mandible | 1.89 | 1.15 | 10.6 | 0.24 | Delayed Implant | 760 |
| 33 | 45 | Half-guided | Mandible | 1.28 | 1.29 | 2.26 | 1.67 | Delayed Implant | 980 |
| 34 | 47 | Half-guided | Mandible | 0.87 | 0.64 | 7.1 | 0.31 | Delayed Implant | 750 |
| 35 | 32 | Half-guided | Mandible | 1.08 | 1.04 | 1.63 | 1.04 | Immediate Implant | 850 |
| 36 | 42 | Half-guided | Mandible | 1.02 | 1.09 | 0.15 | 1.03 | Immediate Implant | 670 |
| 37 | 37 | Half-guided | Mandible | 0.7 | 0.69 | 1 | 0.69 | Delayed Implant | 1210 |
| 38 | 46 | Half-guided | Mandible | 1.31 | 1.14 | 9.15 | 2.09 | Delayed Implant | 870 |
| 39 | 47 | Half-guided | Mandible | 1.03 | 1.13 | 4.02 | 1.69 | Delayed Implant | 920 |
| 40 | 31 | Half-guided | Mandible | 0.66 | 0.6 | 1.02 | 0.6 | Delayed Implant | 970 |
| 41 | 42 | Half-guided | Mandible | 1.53 | 1.52 | 0.36 | 1.54 | Delayed Implant | 860 |
| 42 | 31 | Half-guided | Mandible | 1.05 | 0.67 | 3.06 | 0.62 | Delayed Implant | 1250 |
| 43 | 42 | Half-guided | Mandible | 0.58 | 0.73 | 1.38 | 0.58 | Delayed Implant | 1175 |
| 44 | 31 | Half-guided | Mandible | 1.3 | 1.34 | 4.62 | 1.19 | Delayed Implant | 1075 |
| 45 | 36 | Half-guided | Mandible | 1.39 | 1.49 | 5.85 | 1.38 | Delayed Implant | 680 |
| 46 | 42 | Half-guided | Mandible | 1.53 | 1.47 | 3.93 | 1.07 | Delayed Implant | 1050 |
| 47 | 45 | Half-guided | Mandible | 1.68 | 1.73 | 13.48 | 1.07 | Immediate Implant | 850 |
| 48 | 46 | Half-guided | Mandible | 2.29 | 2.29 | 7.1 | 2.22 | Delayed Implant | 810 |
| 49 | 34 | Half-guided | Mandible | 2.2 | 2.07 | 6.12 | 1.85 | Delayed Implant | 890 |
| 50 | 36 | Half-guided | Mandible | 1.42 | 1.38 | 1.25 | 1.4 | Delayed Implant | 680 |
| 51 | 44 | Half-guided | Mandible | 0.94 | 0.97 | 3.32 | 0.96 | Delayed Implant | 530 |
| 52 | 46 | Half-guided | Mandible | 0.75 | 0.74 | 0.4 | 0.72 | Delayed Implant | 400 |
| 53 | 12 | Full-guided | Maxilla | 0.08 | 0.06 | 1.41 | 0.95 | Delayed Implant | 1050 |
| 54 | 14 | Full-guided | Maxilla | 1.22 | 1.25 | 2.85 | 1.06 | Delayed Implant | 950 |
| 55 | 16 | Full-guided | Maxilla | 1.65 | 2.06 | 6.87 | 1.7 | Delayed Implant | 450 |
| 56 | 17 | Full-guided | Maxilla | 0.79 | 0.8 | 7.59 | 0.74 | Delayed Implant | 360 |
| 57 | 22 | Full-guided | Maxilla | 0.05 | 0.96 | 1.93 | 0.29 | Delayed Implant | 980 |
| 58 | 24 | Full-guided | Maxilla | 1.18 | 1.95 | 4.76 | 1.05 | Delayed Implant | 720 |
| 59 | 26 | Full-guided | Maxilla | 1.16 | 1.16 | 2.51 | 1.1 | Delayed Implant | 468 |
| 60 | 27 | Full-guided | Maxilla | 0.88 | 1.31 | 7.97 | 0.76 | Delayed Implant | 552 |
| 61 | 41 | Full-guided | Mandible | 0.75 | 0.42 | 3.1 | 0.34 | Delayed Implant | 1100 |
| 62 | 46 | Full-guided | Mandible | 1.81 | 1.78 | 7.41 | 1.38 | Delayed Implant | 840 |
| 63 | 36 | Full-guided | Mandible | 1.73 | 2.08 | 7.12 | 0.64 | Delayed Implant | 950 |
| 64 | 17 | Full-guided | Maxilla | 1.26 | 1.27 | 4.08 | 0.31 | Delayed Implant | 346 |
| 65 | 15 | Full-guided | Maxilla | 1.08 | 1.13 | 1.08 | 0.97 | Delayed Implant | 614 |
| 66 | 13 | Full-guided | Maxilla | 0.27 | 0.39 | 2.15 | 0.46 | Delayed Implant | 662 |
| 67 | 22 | Full-guided | Maxilla | 1.79 | 1.35 | 2.04 | 0.53 | Delayed Implant | 576 |
| 68 | 25 | Full-guided | Maxilla | 1.62 | 0.53 | 0.87 | 0.58 | Delayed Implant | 942 |
| 69 | 27 | Full-guided | Maxilla | 1.37 | 1.53 | 4.46 | 0.24 | Delayed Implant | 316 |
| 70 | 16 | Full-guided | Maxilla | 1.39 | 1.36 | 0.75 | 0.71 | Delayed Implant | 564 |
| 71 | 14 | Full-guided | Maxilla | 0.73 | 0.87 | 2.87 | 0.98 | Delayed Implant | 722 |
| 72 | 22 | Full-guided | Maxilla | 0.53 | 1.73 | 2.49 | 0.48 | Delayed Implant | 436 |
| 73 | 24 | Full-guided | Maxilla | 0.83 | 2.39 | 1.99 | 1.04 | Delayed Implant | 584 |
| 74 | 27 | Full-guided | Maxilla | 1.03 | 1.06 | 1.86 | 0.89 | Delayed Implant | 466 |
| 75 | 35 | Full-guided | Mandible | 1.19 | 1.48 | 3.18 | 1.42 | Delayed Implant | 1268 |
| 76 | 32 | Full-guided | Mandible | 1.12 | 0.89 | 1.58 | 1.03 | Delayed Implant | 988 |
| 77 | 42 | Full-guided | Mandible | 1.74 | 1.82 | 3.35 | 1.61 | Delayed Implant | 1066 |
| 78 | 45 | Full-guided | Mandible | 0.93 | 0.17 | 2.83 | 0.84 | Delayed Implant | 1167 |
| 79 | 11 | Full-guided | Maxilla | 0.19 | 0.21 | 1.14 | 0.20 | Immediate Implant | 850 |
| 80 | 23 | Full-guided | Maxilla | 0.73 | 0.95 | 0.6 | 0.71 | Immediate Implant | 1100 |
| 81 | 24 | Full-guided | Maxilla | 1.15 | 1.54 | 2.85 | 0.80 | Immediate Implant | 760 |
| 82 | 32 | Full-guided | Mandible | 0.51 | 0.87 | 0.94 | 1.74 | Immediate Implant | 980 |
| 83 | 34 | Full-guided | Mandible | 0.29 | 1.79 | 2.97 | 0.52 | Immediate Implant | 750 |
| 84 | 36 | Full-guided | Mandible | 1.04 | 1.25 | 6.1 | 0.66 | Immediate Implant | 850 |
| 85 | 42 | Full-guided | Mandible | 0.12 | 0.57 | 1.92 | 0.01 | Immediate Implant | 670 |
| 86 | 44 | Full-guided | Mandible | 0.48 | 0.44 | 0.26 | 0.14 | Immediate Implant | 1210 |
| 87 | 46 | Full-guided | Mandible | 0.87 | 0.55 | 4.21 | 0.55 | Immediate Implant | 870 |

**Table S2**

Detailed analysis of characteristic parameters of full-guided and half-guided surgical templates in different groups

| Maxilla | Template Restriction Type |  | t/U | p |
| --- | --- | --- | --- | --- |
| Coronal Deviation | Half-guided | 1.12±0.63 | 0.838 | 0.411 |
|  | Full-guided | 0.95±0.5 |  |  |
| Apical Deviation | Half-guided | 1.33（0.61，2） | 0.314 | 0.327 |
|  | Full-guided | 1.21（0.85，1.53） |  |  |
| Angular Deviation | Half-guided | 4.67（2.02，13.34） | 2.012 | 0.045^*^ |
|  | Full-guided | 2.32（1.34，4.18） |  |  |
| Depth Deviation | Half-guided | 0.91±0.59 | 0.995 | 0.327 |
|  | Full-guided | 0.75±0.36 |  |  |
| Bone Density | Half-guided | 729.29±138.66 | 1.023 | 0.313 |
|  | Full-guided | 657.64±236.71 |  |  |
| Mandible | Template Restriction Type |  | t/U | p |
| Coronal Deviation | Half-guided | 1.22±0.49 | 1.577 | 0.121 |
|  | Full-guided | 0.97±0.55 |  |  |
| Apical Deviation | Half-guided | 1.2±0.49 | 0.669 | 0.507 |
|  | Full-guided | 1.09±0.65 |  |  |
| Angular Deviation | Half-guided | 3.19（1.21，5.92） | 0.043 | 0.966 |
|  | Full-guided | 3.1（1.75，5.16） |  |  |
| Depth Deviation | Half-guided | 1.21±0.62 | 1.938 | 0.058 |
|  | Full-guided | 0.84±0.56 |  |  |
| Bone Density | Half-guided | 891.45±208.25 | 1.329 | 0.19 |
|  | Full-guided | 977.62±180.53 |  |  |
| Anterior Teeth | Template Restriction Type |  | t/U | p |
| Coronal Deviation | Half-guided | 1.07（0.74，1.53） | 2.181 | 0.029^*^ |
|  | Full-guided | 0.52（0.14，1.03） |  |  |
| Apical Deviation | Half-guided | 1.09±0.44 | 1.302 | 0.204 |
|  | Full-guided | 0.85±0.56 |  |  |
| Angular Deviation | Half-guided | 1.59（1.08，4.1） | 0.127 | 0.899 |
|  | Full-guided | 1.93（1.21，2.41） |  |  |
| Depth Deviation | Half-guided | 1.09±0.48 | 2.085 | 0.046^*^ |
|  | Full-guided | 0.7±0.54 |  |  |
| Bone Density | Half-guided | 937.22±188.88 | 0.859 | 0.398 |
|  | Full-guided | 871.5±228.27 |  |  |
| Posterior Teeth | Template Restriction Type |  | t/U | p |
| Coronal Deviation | Half-guided | 1.25±0.57 | 0.967 | 0.338 |
|  | Full-guided | 1.12±0.38 |  |  |
| Apical Deviation | Half-guided | 1.23（0.91，1.8） | 0.146 | 0.884 |
|  | Full-guided | 1.27（0.87，1.78） |  |  |
| Angular Deviation | Half-guided | 5.32（1.43，9.51） | 1.366 | 0.172 |
|  | Full-guided | 2.97（1.99，6.1） |  |  |
| Depth Deviation | Half-guided | 1.16±0.69 | 2.058 | 0.044^*^ |
|  | Full-guided | 0.83±0.38 |  |  |
| Bone Density | Half-guided | 800.44±198.24 | 1.173 | 0.246 |
|  | Full-guided | 726.91±275.22 |  |  |
| Tooth-Supported | Template Restriction Type |  | t/U | p |
| Coronal Deviation | Half-guided | 1.09±0.45 | 1.355 | 0.18 |
|  | Full-guided | 0.93±0.48 |  |  |
| Apical Deviation | Half-guided | 1.17（0.67，1.44） | 0.347 | 0.729 |
|  | Full-guided | 1.1（0.56，1.52） |  |  |
| Angular Deviation | Half-guided | 2.65（1.37，5.62） | 1.13 | 0.26 |
|  | Full-guided | 2.1（1.1，3.13） |  |  |
| Depth Deviation | Half-guided | 1.09±0.65 | 2.473 | 0.016^*^ |
|  | Full-guided | 0.73±0.44 |  |  |
| Bone Density | Half-guided | 863.26±198.79 | 1.413 | 0.163 |
|  | Full-guided | 781.54±271.06 |  |  |
| Mucosa-Supported | Template Restriction Type |  | t/U | p |
| Coronal Deviation | Half-guided | 1.5±0.51 | 1.875 | 0.077 |
|  | Full-guided | 1.03±0.6 |  |  |
| Apical Deviation | Half-guided | 1.5±0.49 | 0.896 | 0.382 |
|  | Full-guided | 1.26±0.67 |  |  |
| Angular Deviation | Half-guided | 5.12±3.83 | 0.177 | 0.862 |
|  | Full-guided | 4.87±2.57 |  |  |
| Depth Deviation | Half-guided | 1.32±0.47 | 2.06 | 0.054 |
|  | Full-guided | 0.91±0.42 |  |  |
| Bone Density | Half-guided | 773.89±224.83 | 0.075 | 0.941 |
|  | Full-guided | 765.45±266.95 |  |  |
| Immediate Implant | Template Restriction Type |  | t/U | p |
| Coronal Deviation | Half-guided | 1.11±0.34 | 2.932 | 0.01^**^ |
|  | Full-guided | 0.6±0.37 |  |  |
| Apical Deviation | Half-guided | 1.16（0.65，1.56） | 0.096 | 0.923 |
|  | Full-guided | 0.87（0.50，1.40） |  |  |
| Angular Deviation | Half-guided | 1.47（0.57，10.39） | 2.311 | 0.021^*^ |
|  | Full-guided | 1.92（0.77，3.59） |  |  |
| Depth Deviation | Half-guided | 1.12±0.48 | 0.854 | 0.407 |
|  | Full-guided | 0.91±0.53 |  |  |
| Bone Density | Half-guided | 912.5±133.71 | 0.252 | 0.805 |
|  | Full-guided | 893.33±174.28 |  |  |
| Delayed Implant | Template Restriction Type |  | t/U | p |
| Coronal Deviation | Half-guided | 1.17±0.51 | 0.671 | 0.505 |
|  | Full-guided | 1.08±0.5 |  |  |
| Apical Deviation | Half-guided | （0.73，1.51） | 0.11 | 0.91 |
|  | Full-guided | （0.85，1.74） |  |  |
| Angular Deviation | Half-guided | （1.4，5.84） | 0.36 | 0.72 |
|  | Full-guided | （1.91，4.54） |  |  |
| Depth Deviation | Half-guided | 1.14±0.64 | 2.103 | 0.039^*^ |
|  | Full-guided | 0.85±0.4 |  |  |
| Bone Density | Half-guided | 836.02±213.43 | 1.676 | 0.098 |
|  | Full-guided | 736.04±282.56 |  |  |

*P<0.05, **P<0.01

**Table S3**

**Power calculation and effect size results**

| No. | Parameter | Comparison Group | Cohen's d | Interpretation | n (H/F) | Power (1-β) | Clinical Significance |
| --- | --- | --- | --- | --- | --- | --- | --- |
| Overall Comparisons | | | | | | | |
| 1 | Coronal Deviation | Overall | 0.461 | Medium | 52/35 | 0.551 | Moderate evidence |
| 2 | Apical Deviation | Overall | 0.267 | Small | 52/35 | 0.227 | Insufficient evidence ⚠️ |
| 3 | Angular Deviation | Overall | 0.493 | Medium | 52/35 | 0.606 | Moderate evidence |
| 4 | Depth Deviation | Overall | 0.651 | Medium-Large | 52/35 | 0.837 | Strong evidence ✓ ✓ |
| Subgroup Analyses by Anatomical Location | | | | | | | |
| 5 | Angular Deviation | Maxilla | 0.351 | Small-Medium | 14/22 | 0.17 | Weak evidence ⚠️ |
| Subgroup Analyses by Tooth Position | | | | | | | |
| 6 | Coronal Deviation | Anterior | 0.794 | Medium-Large | 16/12 | 0.516 | Moderate evidence |
| 7 | Depth Deviation | Anterior | 0.705 | Medium-Large | 16/12 | 0.428 | Moderate evidence |
| 8 | Depth Deviation | Posterior | 0.613 | Medium-Large | 36/23 | 0.617 | Moderate-Strong evidence |
| Subgroup Analyses by Support Type | | | | | | | |
| 9 | Depth Deviation | Tooth-supported | 0.649 | Medium-Large | 38/24 | 0.687 | Moderate-Strong evidence |
| Subgroup Analyses by Implantation Timing | | | | | | | |
| 10 | Coronal Deviation | Immediate | 1.525 | Large | 8-Aug | 0.81 | Very strong evidence ✓✓ |
| 11 | Angular Deviation | Immediate | 0.491 | Medium | 8-Aug | 0.15 | Weak evidence ⚠️ |
| 12 | Depth Deviation | Delayed | 0.521 | Medium | 44/27 | 0.556 | Moderate evidence |

| Note: H = Half-guided; F = Full-guided |  |
| --- | --- |
| ✓✓ = Power ≥ 0.80 (Adequate) |  |
| ✓ = Power 0.70-0.79 (Acceptable) |  |
| ⚠️ = Power < 0.50 (Underpowered, interpret with caution) | |
